# Seven Golden Rules for heuristic filtering of molecular formulas obtained by accurate mass spectrometry

**DOI:** 10.1186/1471-2105-8-105

**Published:** 2007-03-27

**Authors:** Tobias Kind, Oliver Fiehn

**Affiliations:** 1 University of California Davis, Genome Center, 451 E. Health Sci. Dr., Davis, CA 95616, USA

## Abstract

**Background:**

Structure elucidation of unknown small molecules by mass spectrometry is a challenge despite advances in instrumentation. The first crucial step is to obtain correct elemental compositions. In order to automatically constrain the thousands of possible candidate structures, rules need to be developed to select the most likely and chemically correct molecular formulas.

**Results:**

An algorithm for filtering molecular formulas is derived from seven heuristic rules: (1) restrictions for the number of elements, (2) LEWIS and SENIOR chemical rules, (3) isotopic patterns, (4) hydrogen/carbon ratios, (5) element ratio of nitrogen, oxygen, phosphor, and sulphur versus carbon, (6) element ratio probabilities and (7) presence of trimethylsilylated compounds. Formulas are ranked according to their isotopic patterns and subsequently constrained by presence in public chemical databases. The seven rules were developed on 68,237 existing molecular formulas and were validated in four experiments. First, 432,968 formulas covering five million PubChem database entries were checked for consistency. Only 0.6% of these compounds did not pass all rules. Next, the rules were shown to effectively reducing the complement all eight billion theoretically possible C, H, N, S, O, P-formulas up to 2000 Da to only 623 million most probable elemental compositions. Thirdly 6,000 pharmaceutical, toxic and natural compounds were selected from DrugBank, TSCA and DNP databases. The correct formulas were retrieved as top hit at 80–99% probability when assuming data acquisition with complete resolution of unique compounds and 5% absolute isotope ratio deviation and 3 ppm mass accuracy. Last, some exemplary compounds were analyzed by Fourier transform ion cyclotron resonance mass spectrometry and by gas chromatography-time of flight mass spectrometry. In each case, the correct formula was ranked as top hit when combining the seven rules with database queries.

**Conclusion:**

The seven rules enable an automatic exclusion of molecular formulas which are either wrong or which contain unlikely high or low number of elements. The correct molecular formula is assigned with a probability of 98% if the formula exists in a compound database. For truly novel compounds that are not present in databases, the correct formula is found in the first three hits with a probability of 65–81%. Corresponding software and supplemental data are available for downloads from the authors' website.

## Background

### 1.1 Structure elucidation utilizes NMR and MS

Since more than 50 years mass spectrometric techniques are utilized to identify unknown compounds. Pioneers of structure elucidation around Carl Djerassi [1] or natural product researchers like Satoshi Omura [2] or Victor Wray [3] often used additional analytical techniques like nuclear magnetic resonance, Fourier-transform infrared spectroscopy, ultraviolet spectroscopy or crystallographic data for initial proposition of structures of natural products which were subsequently confirmed by organic synthesis. The famous Dendral project [4] was one of the first concerted actions for structure elucidation approaches using computers which led to the term CASE (Computer-Assisted Structure Elucidation). Machine learning techniques (at this time called Artificial Intelligence), heuristic rules [4] and other chemometrical methods were combined to investigate nuclear magnetic resonance (NMR) and mass spectrometry (MS) data in order to find the correct structure of unknown chemicals in shorter time than manual investigation of all spectral data. Although microflow-NMR [5] was introduced recently using new capNMR probes or cryogenic probes, NMR still lacks sensitivity compared to MS. Two dimensional NMR coupling experiments need measurement times up to hours. More importantly, complex mixtures cannot be fully resolved by NMR data acquisition only. For this purpose, NMR is coupled to high pressure liquid chromatography (HPLC) to separate mixtures prior to data acquisition. Data acquired solely by HPLC-^1^H-NMR are insufficient for *de novo *structure elucidation purposes [6]. Only if separation of co-eluting compounds is complete, resulting in a high enough mass of a pure compound, NMR couplings of protons, carbons and heteronuclei can in principle be used for a full structure elucidation, even without mass spectral data [7]. However, a rapid and automated annotation and structure elucidation of small molecules is still a challenge for complex mixtures. Due to its universality and sensitivity, mass spectrometry is a method of choice as starting point for identification procedures. Library search strategies using the fragmentation pattern of mass spectra of known and available compounds are well established [8]. However, small molecules can not be sequenced like peptides or proteins [9]; hence there is no universal approach for de-novo structure elucidation utilizing MS/MS or MS^n ^techniques for unknown compounds or for the 30 million currently known isomeric structures that are not commercially available.

Separation techniques like HPLC or ultrahigh pressure liquid chromatography (UPLC), capillary electrophoresis (CE) or gas chromatography (GC) are routinely coupled to mass spectrometry to separate and eventually detect and identify all components of complex matrices. Yet reaching such comprehensive aims is far from reality. For the identification of an unknown compound by mass spectrometry, first and foremost a correct elemental composition of the native chemical must be computed. We have recently published an approach how to utilize isotopic abundance patterns of mass spectra for the reduction of formula candidates [10] and how public databases can be searched and compounds can be annotated by their molecular formula. We here extend this approach by establishing and implementing chemical and heuristic rules that are used as constraints for finding the correct chemical formula, and we validate this approach on a large database consisting of 432,968 molecular formulas which covered a chemical space of more than five million compounds.

Several other programs have been suggested in the past for this purpose but none of them was validated on larger datasets like in this study. One program directly uses electron impact (EI) spectra and performs a compatibility check of the molecular formula on EI mass spectra [11]. For that purpose fragmentation patterns are examined and molecular formulas are calculated for these fragments using additional constraints. Another suite of programs used a test set of 900 compounds for interpretation of low resolution mass spectra and computation of highly probable elemental compositions [12]. Subsequently, a set of empirical filter functions is used, but the validity of these filters was not thoroughly investigated due to the limited size of the molecular test sets.

### 1.2 Compound identification requires high resolution and high accuracy of mass and isotope ratio measurements

In this paper we assume compounds to be completely resolved from co-eluting or isobaric compounds by the combination of chemical separation and high mass resolution [13]. Ultrahigh mass accuracy (less than 1 ppm) and high resolving power (750,000 at m/z 400; FWHM, full width at half maximum) can be obtained with Fourier-transform ion cyclotron mass spectrometry (FT-MS) [14] or by Orbitrap mass spectrometers [15] (max. resolving power 100,000; FWHM). However, data acquired even by ultrahigh mass accuracy and mass resolution are insufficient for calculating unique elemental compositions without information about isotope ratios [10]. Isotope ratios are measured since the very beginning of mass spectrometry [16]. Natural occurring elements can be monoisotopic (F, Na, P, I) or polyisotopic (H, C, N, O, S, Cl, Br) [17]. The calibrated abundance values of these elements are reported by the IUPAC [18]. Using isotopic pattern generators [19] one can calculate the contribution to the abundances of the M+1, M+2, M+3 isotope ions in mass spectra, where M^+• ^or M^-• ^reflect the molecular ion. As the number of elements increase in complex and large molecules, the computation of correct isotope ratios becomes more complicated [20]. In fact, small molecule formulas can be calculated even from low-resolution quadrupole mass spectrometry data when isotope patterns and silylations were included as search constraints [21].

### 1.3 Raw data processing for complex samples involves peak picking, mass spectral deconvolution, and determination of molecular ions by adduct detection

Even techniques with high peak capacities such as GCxGC/MS (comprehensive GC) or UPLC/MS will lead to partially co-eluting peaks for complex mixtures. Moreover, low abundant compounds may not be apparent by visual inspection of chromatograms. Detection of single components from complex chromatograms is therefore performed by peak picking and mathematical deconvolution routines. The development of the freely available AMDIS program [22] was a major milestone for GC/MS data analysis. The CODA algorithm [23] for LC/MS data analysis is now implemented into different commercial packages. Such peak picking and mass spectrometric deconvolution routines are obligatory unless chemicals purified otherwise are directly introduced into the mass spectrometer via a probe or direct infusion.

The determination of the molecular ion from a given mass spectrum is one of the crucial steps during mass spectral evaluation. For electron impact spectra (EI) this problem has been tackled [24] and the corresponding algorithm is implemented in the NIST MS-Search Program [25]. Most often, GC/MS instruments are run under EI ionization. Unfortunately, molecular ions observed in EI spectra frequently have very low abundances or are complete absent (specifically when using trimethylsilyl derivatives) which limit the use of molecular ion calculations. Instead, soft ionization techniques like chemical ionization (CI), field emission (FE), field desorption (FD) or other methods may be employed for ionizing compounds separated by GC. Resulting from soft ionization, GC/MS spectra often comprise abundant molecular ions which may be utilized for assigning molecular formulas if an accurate mass spectrometer is used. LC/MS surveys almost exclusively use soft ionization procedures (electrospray or atmospheric pressure chemical ionization), both producing adducts of molecular ions such as [M+H]^+ ^or [M+Na]^+ ^or multiple others. Obviously, the nature of the adduct formation must be determined before accurate mass data acquisitions can be used for assigning elemental compositions [26]. Although the problem is well known since several years, only recently a commercial program has been released to the market [27]. Yet, routine recognition of adduct formation in LC/MS has not been validated on large datasets of diverse mass spectra.

## Results

### Developing rules for constraining formula generators

#### 2.1 The rings-and-double-bond equivalent and the nitrogen rule are not instrumental for calculating sum formulas

Atoms in chemical structures are connected by one or multiple chemical bonds. Consequently, the *rings-plus-double-bonds equivalent *(RDBE) or *double-bond equivalent *(DBE) concept was introduced in the 1950's [28,29], and shortly later, the term of "degree of unsaturation" was introduced [30]. RDBE values can be calculated the following formula [31]:

RDBE = C+Si - 1/2(H+F+Cl+Br+I) + 1/2(N+P)+1     (1)

Each element symbol here represents the count of atoms of this element in the molecular formula. The elements oxygen and sulphur were not taken into account. This equation is still in use by organic chemists and mass spectrometrists, and it is frequently used in mass spectrometry software and common molecular generators. However, the equation is based on the lowest valence state for each element which therefore does not allow an exhaustive and correct calculation of the true RDBE values for a given accurate mass. So even if double bonds exist in the molecule, the formula does not count the number of double bonds correctly. Due to this known problem a more general approach was suggested to calculate the degree of unsaturation which also includes excess localized electrons [32]. However, even this approach does not yield unique solutions. The elements nitrogen and phosphorous can have three or five valences, and sulphur atoms may have two, four or six valences. In organic compounds nitrogen has a valence of three. For molecules that contain these three atoms in different mixed combinations of their valence states, no single solution for RDBE can be calculated but an RDBE range would result.

Moreover, negative RDBE numbers can not be excluded *a priori*, because normal valence state may be exceeded. More than 64 substances were found to have negative RDBE numbers when querying formulas in the NIST and Wiley mass spectral databases. Most of these compounds contained either chlorine or fluorine atoms together with elements N, O, S or P. For example C_12_H_36_F_6_N_6_O_2_P_4_Si_2 _(CAS: 110228-63-2, RDBE = -1) is a substance with phosphorous in the valence of five. CH_2_F_10_S_2 _(CAS: 117146-25-5, RDBE = -4) comprises sulphur at a valence state of six. Organic sulphates contain sulphur at a valence state of six. For example, for dimethyl sulfate an RDBE value of zero is calculated although actually two double bonds are present in the molecular structure. Due to these uncertainties, it cannot be automatically decided if the RDBE value for a specific formula is correct. Therefore, RDBE values are of rather limited use as constraint in assigning chemically possible elemental formulas. However, a helpful application of the rings-plus-double-bonds equivalent is to detect formulas with an extremely high RDBE value. For example, RDBE values may be utilized to either exclude or include fullerenes, like C_78_H_12_Cl_2_N_2 _with an RDBE of 73. Most of the compounds in our test set (99.90%) were found to comprise an RDBE of less than 40.

These results on assumptions and limitations of RDBE values exclude using rings-and-double-bond equivalents as constraint in calculation of molecular formulas. Instead, we have assumed all chemically possible valence values be valid for all elements under investigation, in order to allow an exhaustive calculation of elemental compositions. Consequently, the number of theoretically allowed molecular formulas is dramatically increased in the first instance, reflecting the chemical diversity of molecules that can be built from the scaffold of elements and their oxidation and valence states.

The *nitrogen rule *states that an odd nominal molecular mass implies also an odd number of nitrogens. This rule should only be used with nominal (integer) masses. When using accurate mass measurements this rule becomes unreliable in mass ranges higher than 500 Da. A test performed with 17,000 formulas from 27–3000 Da resulted in 20% wrong assignments for the number of odd or even nitrogens. This is due to the fact that small non-nominal mass contributions from a large number of elements add up in higher mass regions. An example would be C_38_H_64_N_2 _(accurate mass 548.50692 Da; integer mass 548 Da). This rule can be helpful in lower mass ranges using unit resolution mass spectrometers or during assignment of elemental compositions to small fragments.

Below, we demonstrate how a combination of heuristic and chemical rules reduces the number of theoretical formulas to a small set of the most likely compositions. The development and validation for each of the seven rules is shown in the following sections.

#### 2.2 Establishing heuristic and chemical rules

##### Rule #1 – restrictions for element numbers

The restriction of element numbers is important to save computational time and disk space. If the research is aimed towards natural compounds (hence, excluding peptides) there is no reason to include chemicals with unreasonable high element counts. For developing this rule, the absolute element limits were calculated by simply dividing the mass range through the element mass (e.g. for carbon = 12 Da at 1000 Da follows 1000/12 = 83 maximum limit for a hypothetical molecule that consists exclusively of carbon. The maximum element count was further restricted in a heuristic manner by using the development set of formulas that were derived from NIST and Wiley mass spectra and DNP entries. Results are given in Table 1. Maximum element counts were defined for the mass ranges at < 500 Da, < 1000 Da, < 2000 Da, < 3000 Da by taking the higher value found in either of the two development databases. For example, for 47 carbon atoms the maximum hydrogen number does not exceed 150 using the most common six elements (C, H, N, S, O, P and Si) and are usually much lower. For natural products, the numbers for nitrogen, phosphor and sulphur are smaller than those of peptides. For example, in the range 960–1080 Daltons, peptides show a maximum nitrogen number of 36 but for natural small molecules, a maximum of only 30 is found.

**Table 1:** Restrictions for number of elements during formula generation for small molecules based on examination of the DNP and Wiley mass spectral databases. For each element, the higher count was taken for denominating the element restriction rule #1

**Mass Range [Da]**	**Library**	**C max**	**H max**	**N max**	**O max**	**P max**	**S max**	**F max**	**Cl max**	**Br max**	**Si max**
< 500	DNP	29	72	10	18	4	7	15	8	5	
	Wiley	39	72	20	20	9	10	16	10	4	8
< 1000	DNP	66	126	25	27	6	8	16	11	8	
	Wiley	78	126	20	27	9	14	34	12	8	14
< 2000	DNP	115	236	32	63	6	8	16	11	8	
	Wiley	156	180	20	40	9	14	48	12	10	15
< 3000	DNP	162	208	48	78	6	9	16	11	8	

##### Rule #2 – LEWIS and SENIOR check

In principle, elemental formulas can be calculated from accurate mass measurements for any species, ions, radicals or neutralized (uncharged) molecules. Since mass spectrometers can only detect ions (and ion radicals), common formula calculators like CHEFOEG [33] or Hires MS [34] calculate all combinations of elements that result in the correct (measured) mass, disregarding advanced tests for the physical existence of chemical structures, because in gas phase chemistry of radicals and ion radicals, many uncommon and short-lived molecule species may exist (as fragments) that would not be described as stable compounds under natural conditions. Conversely, for the objective to determine the correct formula for natural products, it is reasonable to check if a molecular graph (a chemically existent species) can be built from a specific formula. Two of the fundamental deterministic chemical rules are not obeyed by common formula calculators, the LEWIS and SENIOR rules. These rules can best be tested for neutral compounds, hence ionic species detected in mass spectrometry first need to be neutralized by determining the adduct formation and correcting for it. For example, for obtaining the neutral structure from protonated compounds, a common adduct formed under positive electrospray mass spectrometry conditions, subtracting the proton mass of 1.007825 u from the accurate mass data would be required. Without determining and neutralizing the molecular ion, species that are non-existent would be calculated by common calculators such as C_6_H_16_O_3_. Accordingly, any report on accurate mass measurements should at least detail the ion species (adducts) that were determined in order to enable *post hoc *validations.

In its simplest form, the LEWIS rule demands that molecules consisting of main group elements, especially carbon, nitrogen and oxygen, share electrons in a way that all atoms have completely filled *s, p*-valence shells ('octet rule'). However, free radicals such as 5-hydroxy-2,2-dimethyl-1-Pyrrolidinyloxy (C_6_H_12_NO_2_; CAS: 55482-03-6), and in fact, all nitroso compounds, would not be allowed if the LEWIS rule would be strictly enforced. The LEWIS rule marks such compounds to be odd electron molecules, and the RDBE value would be a non-integer (in this case 1.5). If such radical components need to be checked, the rule has to be disabled within the source code of our script. Furthermore, newer quantum mechanic *ab-initio *calculations have shown that certain hypervalent molecules do not obey the LEWIS rule [35], and therefore we have combined it with a test for the extended SENIOR rule. Senior's theorem [36] requires three essential conditions for the existence of molecular graphs [37]:

i) The sum of valences or the total number of atoms having odd valences is even;

ii) The sum of valences is greater than or equal to twice the maximum valence;

iii) The sum of valences is greater than or equal to twice the number of atoms minus 1.

The SENIOR rule is included in advanced molecular isomer generator such as the commercial MOLGEN [38] or the Deterministic Structure Generator [39], which was built with the help of the free Chemistry Development Kit (CDK) [40]. Both calculators have free test-versions available online. However, certain radicals such as nitroso compounds should be excluded in MOLGEN or need additional preparatory steps. More severe limitations are found that both calculators only take ground state valences into account, i.e. sulphur with two valences, phosphorous with three valences. These elements may have higher valences, and importantly, each element may have different numbers of valences in a specific, chemically allowed molecule. So far, calculators were unable to handle all combinations of mixed valences and higher valence states which would discriminate physically existent molecular formulas if directly applied or implemented into algorithms for filtering formulas by chemical rules. Consequently, the algorithm presented here tests for LEWIS and SENIOR rules by allowing maximum valence states for each element, and presence of mixed valence states for each element within molecular structures.

##### Rule #3 – isotopic pattern filter

**Figure 1:**
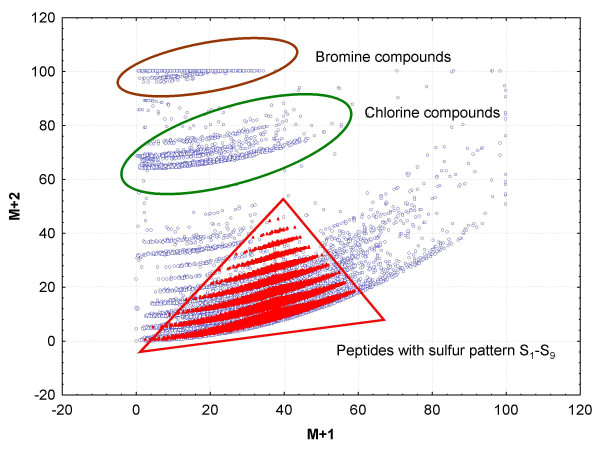
**Isotopic pattern of 45.000 compound formulas from the Wiley mass spectral database and 60.000 peptides formulas in the small molecule space < 1000 Dalton**. M+1 and M+2 are given as relative abundances in [%] and are normalized to 100% of the highest isotope abundance in the molecular formula.

Compounds that were synthesized by natural precursors comprise monoisotopic and isotope masses according to the natural average abundance of stable isotope abundances which are listed for each element [18]. Previously, we have shown that applying isotopic abundance patterns removes most of the wrongly assigned molecular formulas from a certain mass measurement experiment [10], and we proved that without isotope information, even a hypothetical spectrometer capable of 0.1 ppm mass accuracy could not report unique elemental compositions in the range above 185.9760 Dalton when elements C, H, N, S, O and P were included in the search list. Therefore, isotope ratio abundance was included in the algorithm as an additional orthogonal constraint, assuming high quality data acquisitions, specifically sufficient ion statistics and high signal/noise ratio for the detection of the M+1 and M+2 abundances. Mass spectrometers such as time-of-flight instruments are commercially available which report isotopic abundance patterns with a very low relative error (around 2–5% relative standard deviation Figure 1 shows all M+1 and M+2 patterns for the formulas from the Wiley mass spectral database and a data set of calculated peptide formulas. This figure demonstrates that for a few compound classes such as brominated and chlorinated small molecules, but also for sulphur-containing peptides, the isotope pattern is a valuable tool to immediately determine the presence of such elements. For monoisotopic elements (F, Na, P, I) this rule has no impact. The relative impact of the isotopic ratio evaluations against the other rules is highlighted below.

##### Rule #4 – Hydrogen/Carbon element ratio check

**Figure 2:**
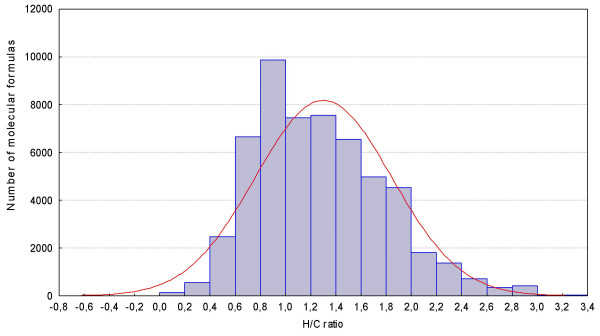
Hydrogen/Carbon ratio (H/C) for 42,000 diverse molecules (containing C, H, N, S, O, P, F, Cl, Br, I, Si) taken from the Wiley mass spectral library.

Another important constraint for restricting formulas to those that are likely to exist is including element ratios, especially the hydrogen/carbon ratio (see Figure 2). In most cases the hydrogen/carbon ratio does not exceed H/C > 3 with rare exception such as in methylhydrazine (CH_6_N_2_). Conversely, the H/C ratio is usually smaller than 2, and should not be less than 0.125 like in the case of tetracyanopyrrole (C_8_HN_5_). Figure 2 demonstrates that most typical ratios are found between 2.0 > H/C > 0.5, for example for long chain alkanes (H/C ~ 2) or polycyclic aromatic hydrocarbons (H/C~ 0.5). Frequency distributions were found to be not Gaussian, so limit ranges could not be defined by 3σ or 4σ standard deviations which would cover 99.7% and 99.99%, resp., of all formulas in the data set. Instead, cumulative percentages were used as range limits, given as Table 2 for examining the Wiley spectral database as development set. More than 99.7% of all formulas were included with H/C ratios between 0.2–3.1. Consequently, we call this range the 'common range'. However, a number of chemical classes fall out of this range, and we have hence enabled the user to select 'extended ranges' covering 99.99% of all formulas in this development database (H/C 0.1–6). There are extreme cases for which rules of typical H/C ratios may be overridden, e.g. for the study of fullerenes [41] which have an extremely low hydrogen/carbon ratio such as in C_78_H_12_Cl_2_N_2_. The implementation of our script enables researchers to exclude certain rules if needed for specific applications.

**Table 2:** Common element ratios obtained from 45.000 formulas comprising the Wiley mass spectral database for the mass range 30 Da – 1500 Da

**Element ratios**	**Common range (covering 99.7%)**	**Extended range (covering 99.99%)**	**Extreme range (beyond 99.99%)**
H/C	0.2–3.1	0.1–6	< 0.1 and 6–9
F/C	0–1.5	0–6	> 1.5
Cl/C	0–0.8	0–2	> 0.8
Br/C	0–0.8	0–2	> 0.8
N/C	0–1.3	0–4	> 1.3
O/C	0–1.2	0–3	> 1.2
P/C	0–0.3	0–2	> 0.3
S/C	0–0.8	0–3	> 0.8
Si/C	0–0.5	0–1	> 0.5

##### Rule #5 – heteroatom ratio check

Element ratio checks strongly reduce the number of candidate formulas. However, we found that heteroatom ratios distributions are even more skewed than H/C ratios, because many formulas comprise no heteroatom at all (such as alkanes) or very few, and rare cases exist with high ratios of heteroatoms to carbon numbers. Table 2 lists the common, the extended and the extreme ratios for small organic compounds comprised in the Wiley mass spectral database.

##### Rule #6 – element probability check

Rule #5 only restricts unlikely high element ratios in molecular formulas, but it does not test for multiple high element counts like in the example of C_26_H_28_N_17_O_1_P_3_S_8_. This formula would pass all rules so far including the element ratio checks; however, the combination of high element ratios would still be too improbable. Therefore, an additional constraint "multiple element count" (see Table 3) was included. All combinations for NOPS and all triple combinations were included in this sub-rule. In order to have a sufficiently large basis for evaluating combinations of element ratios, this sub-rule was developed using the Dictionary of Natural Products and the two mass spectral libraries NIST02 and the Wiley. For element combinations of N, O, P and N, O, S a high number of formulas was found with high element ratios, and specific thresholds were defined accordingly.

**Table 3:** Multiple element count restriction for compounds < 2000 Da, based on the examination of the Beilstein database and the Dictionary of Natural Products

**Element counts**	**Heuristic Rule**	**DB examples for maximum values**
NOPS all > 1	N< 10, O < 20, P < 4, S < 3	C_15_H_34_N_9_O_8_PS, C_22_H_44_N_4_O_14_P_2_S_2_, C_24_H_38_N_7_O_19_P_3_S
NOP all > 3	N < 11, O < 22, P < 6	C_20_H_28_N_10_O_21_P_4_, C_10_H_18_N_5_O_20_P_5_
OPS all > 1	O < 14, P < 3, S < 3	C_22_H_44_N_4_O_14_P_2_S_2_, C_16_H_36_N_4_O_4_P_2_S_2_
PSN all > 1	P < 3, S < 3, N < 4	C_22_H_44_N_4_O_14_P_2_S_2_, C_16_H_36_N_4_O_4_P_2_S_2_
NOS all > 6	N < 19 O < 14 S < 8	C_59_H_64_N_18_O_14_S_7_

##### Rule #7 – TMS check

Analysis of small molecules is often performed by GC/MS, frequently requiring chemical derivatization of the original molecules to enhance volatility, stability or sensitivity of detection. For applications in metabolomics or clinical chemistry, a commonly used derivatization step involves trimethylsilylation by MSTFA (NMethyltrimethylsilyltrifluoroacetamide; CAS: 24589-78-4) which exchanges acidic protons against TMS (trimethylsilyl) groups. If ionization conditions and molecular structures allow for the observation of molecular ions, TMS groups (C_3_H_8_Si) have to be subtracted for calculating the underivatized molecule. For example, accurate masses for C_20_H_58_N_6_O_4_Si_6 _and C_24_H_62_O_6_Si_6_differ only by 4 ppm, but after subtraction of TMS groups, the residual (native) molecule C_6_H_14_O_6 _is much more likely according to rules #4–6 than C_2_H_10_N_6_O_4_. Both compounds would bear six TMS groups which may mask differences in mass spectral isotope ratios given the high isotope abundances of silicon. Nevertheless, the number of TMS groups can easily be deduced by calculating isotope abundances, as we have shown in earlier work [21]. For TMS derivatized molecules detected in GC/MS analyses, the rules on element ratio checks and valence tests are hence best applied after TMS groups are subtracted, in a similar manner as adducts need to be first recognized and subtracted in LC/MS analyses.

#### 2.4 Combination of all rules

The application of all rules together is sufficient to derive the most likely elemental formula from accurate mass and isotopic ratio mass spectral measurements. In any case, adduct ions (in LC/MS) or TMS-derivatives (in GC/MS) have to be determined [26] in order to obtain the neutral form for each molecule. The seven rules are summarized below.

Seven rules for molecular formula filtering for non-charged molecules:

1) apply heuristic restrictions for number of elements during formula generation

2) perform LEWIS and SENIOR check

3) perform isotopic pattern filter

4) perform H/C ratio check (hydrogen/carbon ratio)

5) perform NOPS ratio check (N, O, P, S/C ratios)

6) perform heuristic HNOPS probability check (H, N, O, P, S/C high probability ratios)

7) perform -TMS check (for GC-MS if a silylation step is involved)

The rules are implemented in an automated script written in Visual Basic and C++ which can also be used to calculate and subtract 45 usual adduct ions in LC/MS applications [26]. The adduct removal must be done manually before entering any values. The script performs all the filtering in an automatic mode, triggered by the user. If formulas are not obeying the rules, they are marked with "NO", whereas allowed molecular formulas are marked with "YES". In addition, the isotopic pattern filter uses the experimental isotopic ratios to assign a score for each formula from 0–100 where higher numbers mean a higher probability of existence. Mass accuracies should be determined for specific conditions, since mass errors are known to be depending on ion statistics, automated versus manual runs, or direct infusion of purified compounds versus LC/MS runs of complex samples. Default values may be used as given by the instrument vendors. In addition to ranking formulas, these formulas are queried against internal target databases containing specific sets of molecular formulas.

The highest ranked molecular formula candidates are directly linked online to the freely available Chemical Structure Lookup Service (CSLS) [42] which covers more than 27 million unique structures from 80 public and commercial databases at the time of writing. The CSLS also links to PubChem [43] but has the advantage of a much faster response time. The Dictionary of Natural Products and other copyrighted databases are searched in-house only.

We have validated the seven rules based on formula compilations downloaded from PubChem in early 2006 (432,968 formulas) and additionally we downloaded and included a peptide database of 120,000 molecular formulas [44] comprising all combinations of the 20 proteinogenic amino acids for masses below 1000 Da.

### 3. Validation and exemplary application of the seven rules

#### 3.1 Compounds in PubChem are covered by the rule constraints

**Figure 3:**
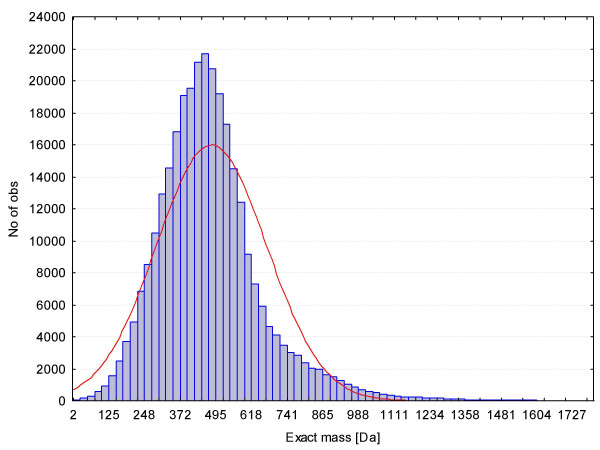
Frequency distribution for the molecular masses of all elemental compositions downloaded from the PubChem database (2006) covering more than 5 million single compounds.

As stated above, rules were evolved from the Wiley mass spectral libraries and the DNP database (~ 47,000 and ~ 31,000 unique elemental compositions, respectively and combined a total of 68,237 unique formulas). The NIST02 mass spectral database was additionally used for special test cases. The mass spectral databases were selected for rule development because mass spectrometry is usually used for molecular formula determination. For validation, we have compiled a much larger database of 432,968 unique molecular formulas that were derived from 5 million compounds from the PubChem database (version February 2006). At the time of writing the PubChem database has almost doubled to 10 million compounds. Even if such queries were possible, online-query times would be rather slow. We have therefore downloaded or purchased the databases for in-house use and rapid access. The PubChem database was found to well represent the known molecular space of small molecules. 27% of all elemental compositions were found between 400–500 Dalton (see Figure 3). Out of a total of more than five million chemical structures, 99.6% of all formulas comprised less than 99 carbon or hydrogen atoms, rendering this number a reasonable cut off that we have utilized in our script implementation. Interestingly, only 2665 molecular formulas (0.6%) failed to pass the set of seven rules we have developed. Among them 1589 molecular formulas did not pass the Lewis and Senior check, either because of conversion errors, wrong formulas or they were true radical containing compounds. Most of the other failed candidates (1481) did not pass rule #6 – the HNOPS probability check – due to the fact that they did not contain any hydrogen. The following largest group (1349 candidates) did not pass rule #4, the hydrogen/carbon ratio test. An overlap analysis revealed that 21,783 molecular formulas (5%) from Wiley and DNP were not contained in the PubChem validation set. These results demonstrate the good performance of the set of rules, despite the comparatively small size of the development databases.

#### 3.2 The space of chemically possible formulas is reduced 13-fold by rules #4–6

The next validation step aimed at assessing the ability of the seven rules to minimize the number of all molecular formulas with less than 2000 Da in mass. We have performed an exhaustive calculation of all formulas in this mass range containing the elements C, H, N, O, S and P which was just constrained by chemical consistency using the LEWIS and SENIOR checks, allowing the maximum valence state for each element. Table 4 lists the result of this validation effort. Without any restrictions, i.e. by using the original HiRes formula generator and its count function, eight billion molecular formulas were generated in 29 hours computational time. Conversely, when using the newly developed brute force formula generator HR2 applying the element count, ratio and probability restrictions, the computational time was reduced to 455 seconds resulting in only 623 million formulas. Using the standard valence of three for nitrogen in organic compounds will slightly reduce the number to 598 million molecular formulas. This 13-fold reduction of formulas validates the use of elemental constraints, especially when novel molecules are to be annotated that are not included in the currently available databases. For example, from each of the most likely formulas, chemical structure generators may be utilized to evolve all structural isomers in either deterministic or stochastic manners, which may subsequently be sorted using other physicochemical constraints. Such exhaustive isomer generations have previously been employed for small molecule research and drug screening [45], and it might become a viable option for cluster computing as search tool in metabolomics. Molecular isomer generators can generate a plethora of structural isomers from one single molecular formula [46]. Obviously, such structure generators can only be successfully applied if the number of input formulas is very small, at best restricted to one single elemental composition.

**Table 4:** Results for number of molecular formulas in ranges < 500, < 1000 and < 2000 Da for elements CHNSOP with maximum valencies (v_N _= 5, v_S _= 6, v_P _= 5) and maximum element counts, last column with element count restrictions from either DNP or Wiley database

Mass range (u)	Maximum number of molecular formulas	With element ratio check	With probability check	With probability check + element count restriction
500	2,707,540	1,772,483	729,617	724,270
1000	139,735,355	87,888,303	32,555,050	30,077,741
2000	7,995,776,805	4,926,973,096	1,170,870,061	623,270,049

#### 3.3 Validation by simulated mass spectral data of 6,000 chemically diverse compounds

We have therefore further explored the validity of the seven rules by simulating data acquisition errors that were imposed on subsets of compounds from four important application databases: the Dictionary of Natural Products, the open access DrugBank database [47], the Toxic Substances Control Act database (TSCA) and compounds from the NIST and Wiley mass spectral libraries. Compound subsets were selected randomly except for the mass spectral library entries which were chosen based on the constraint that formulas were absent from the PubChem, DNP or the peptide libraries. For each database subset, compounds were selected in a way that preserved the original distribution of isotope ratios and mass ranges. For all selected compounds, mass spectral measurements were simulated at ± 3 ppm mass accuracy and with ± 5% isotopic ratio errors. Such performance values should easily be achieved by time-of-flight mass spectrometers. These data were imposed by assumed random errors reflecting mass spectrometric data acquisition, using the normal cumulative distribution in order to introduce noise into the selected datasets.

**Figure 4:**
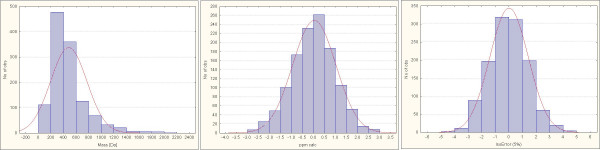
**Frequency distribution for 1,200 randomly selected molecules downloaded from the Dictionary of Natural Products at < 2000 Da and comprising C, H, N, S, O, P, F, Cl and Br. Left panel, 4a: mass distribution**. Middle panel, 4b: simulated measured masses at 3 ppm mass accuracy. Right panel, 4c: simulated measured isotope ratios at ± 5% accuracy.

For each of these targets a ranking was performed applying the set of seven rules, and error rates were determined. Statistics were based on the mass distribution of each of the target libraries. Most of the formulas were found in the range between 300–600 Da (see Figure 4a), and the effect of imposing assumed data acquisition errors is given in Figure 4b and 4c. Table 5 details the result of this validation experiment. It was found that when applying the set of seven rules and querying the correct target database, the correct molecular formula was ranked at the top of the list of potential other formulas in almost all cases (98–99%). If the more general (and much larger) PubChem database was queried, still the correct chemical formula would be retrieved as top candidate in 84–90% of the test cases, and only in 8–10% of the cases, a wrong formula was ranked highest (either due to higher ranking of another PubChem compound or due to absence of the correct formula in PubChem). The residual 2–5% of the cases represents true negatives, i.e. no PubChem formula was retrieved to fit the data because the input formula from the target databases DrugBank, DNP or TSCA were indeed absent in PubChem. Even without using any query of a formula data base, the correct input formula was ranked among the top three candidates in about 80% of the cases by the scoring function. In addition, we have deliberately selected 1200 formulas from the Wiley and NIST mass spectral databases that were absent in PubChem. These truly 'unknown compounds' would be ranked mostly among the top three hits using the set of seven rules alone; however, when querying the PubChem database, a high number of false positive formula annotations would result.

**Table 5:** Validation of the seven rules using random sub-sampled test sets from specialized databases. Performance is given assuming mass spectrometry errors of ± 5% isotope abundance error and ± 3 ppm mass accuracy and calculating element combinations of C, H, N, S, O, P, F, Cl and Br

**Test set and Source**	**Number of random formulas**	**Mass range [Da]**	**target DB top hit [%]**	**PubChem top hit [%]**	**PubChem false top hit [%]**	**no DB query top 3 hits [%]**
Pharmaceuticals (DrugBank)	2400	30–1093	99	90	8	78
Natural Products (DNP)	1200	92–2020	99	84	10	81
Toxic Chemicals (TSCA)	1200	56–2170	98	87	8	78
Unknowns taken from Wiley+NIST	1200	150–1536	-	-	78	65

**Figure 5:**
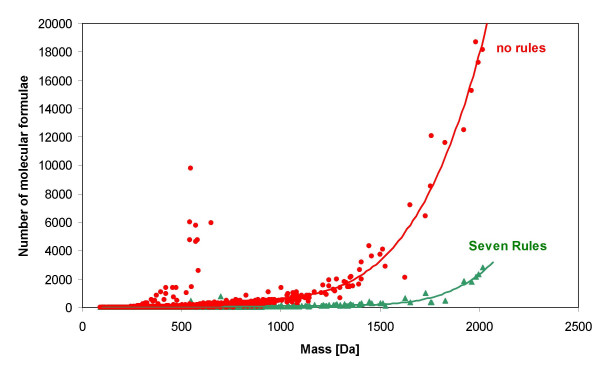
**Mass dependence of calculated, chemically possible formulas derived from 1,200 randomly selected DNP molecules, imposed with simulated 3 ppm mass accuracy ± 5% isotope ratio measurement errors**. Red graph: number of calculated formulas with common molecular generators. Green graph: number of formulas constrained by the seven rules. Outliers around 600 Dalton were found to be halogen containing compounds.

**Figure 6:**
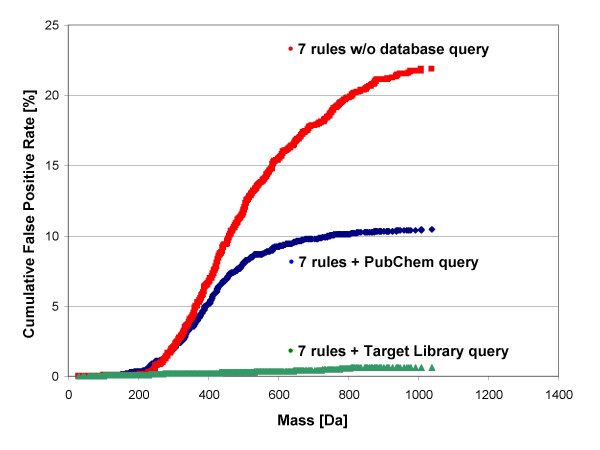
**Effect of ranking the output formulas of the 2,400 randomly selected DrugBank molecules, imposed with simulated ± 3 ppm mass accuracy ± 5% isotope ratio measurement errors**. Mass dependence is shown for no database query (red graph, correct formula found in the top three hits), PubChem database query (blue graph, correct formula ranked top) or querying the DrugBank database (green graph, correct formula ranked top).

This result emphasizes that our algorithm correctly annotates formula if these are present in small molecule databases, but such annotations should not be confused with unambiguous identifications. Instead, genuine compound identifications require additional information such as ion fragmentations [48] or verifications by pure reference compounds. We have further investigated this data set of 6,000 target formula on the mass dependence of the number of generated formula (figure 5) and the number of correct top hits (figure 6), still assuming a 3 ppm error in mass accuracy and 5% isotope ratio error. Figure 5 demonstrates the constraining power of the seven rules at this level of mass spectrometric accuracy. The well-known explosion of chemically possible formulas at higher mass ranges is restricted by around 80% even at 2,000 Da. However, the sole use of the seven rules does not generate unique elemental formulas. Up to about 800 Da, the scoring function alone is sufficient to rank the correct formula at the top without use of database queries with an accuracy of 80%. When querying the large PubChem database, the correct formula is ranked top with an accuracy of about 88% even at extended mass ranges, and when the (smaller) target libraries are applied, the correct formula was retrieved as top hit with a 98% rate even at high masses.

#### 3.4 Example applications of the seven rules

**Figure 7:**
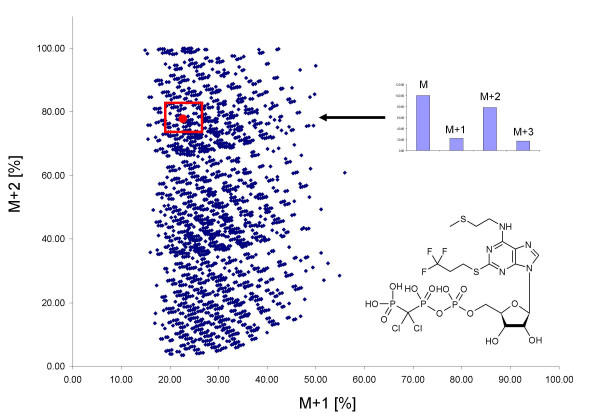
**Relative isotopic abundances of the M+1 and M+2 peak for all elemental compositions that would fit a measured mass of 774.94831 Da (Cangrelor), determined at 1 ppm mass accuracy (values exceeding 100% are removed in graphics)**. Most formulas can be discarded if isotope ratios are measured with an accuracy of ± 5% and used as search constraint (red box).

We exemplify a worst case scenario for molecular formula determination from (theoretical) accurate mass spectrometry data: to annotate the pharmaceutical drug Cangrelor comprising eight different elements (C_17_H_25_Cl_2_F_3_N_5_O_12_P_3_S_2_; CAS: 163706-06-7; 774.94831 Da). Even within an error of +/-0.1 ppm mass accuracy more than 449 elemental compositions are possible. More realistic data acquisition errors are, in fact, in the range of 1 ppm even with advanced mass spectrometers. For Cangrelor, 1 ppm mass accuracy would result in 4,465 molecular formulas to be generated by HR2 in 60 seconds. The isotope ratio rule alone would exclude 4,330 elemental compositions if a 5% isotope accuracy could be assumed, marked by a red box in Figure 7. A score function which matches the experimentally obtained pattern against all theoretical calculated patterns can be used to further rank sum formulas. If an accuracy of 2% relative standard deviation is assumed, only 25 formulas are left when applying all seven rules. The correct formula is the only remaining elemental composition if this rank is queried against the target drug data base (but it was not present in PubChem at the time of search). In other cases, investigation of tandem mass spectra would need to be included as further constraint.

Next, we have taken actual experimental results to validate the approaches using known compounds. First, GC-time-of-flight MS measurement data were taken for which a mass of 615.324 was obtained for the [M+H]^+ ^ion of the sugar alcohol sorbitol under chemical ionization [49]. The neutralized molecule would thus have an adjusted mass of 614.316 which results in 696 elemental compositions if 5 ppm mass accuracy is assumed and the elements C, H, N, O, S, P and Si are considered, based on calculation with the MWTWIN smart hydrogen option and no restriction on element counts. Even if time-of-flight instruments would be improved to empower 1 ppm accurate mass acquisitions, still 148 formulas would be obtained. For this example, the impact of each of the seven rules was investigated, and results are given in Table 6. Isotopic pattern were calculated with a modified Mercury6 version. None of the seven rules alone was efficiently removing wrong elemental compositions but altogether, the set of rules efficiently discarded more than 98% of wrong or unlikely formulas. Among the rules, still rule #3 testing isotope ratios was most efficient, but rule #2, the Senior and Lewis check and rule #7, the TMS check, also proved to be very powerful by alone rejecting 60% and 62%, respectively. Finally the resulting ten formulas were sorted according to their score matching the experimental to calculated isotopic patterns, and the correct formula for Sorbitol was ranked as top candidate.

**Table 6:** Single performance of each rule of the seven rules from a total of 696 formulas comprising the elements CHNSOP and Si, calculated from GC-TOF data of sorbitol TMS_6_

**Rules for molecular formula filtering**	**Single application of each rule**
1) heuristic restrictions for number of elements	not used (smart H option instead)
2) perform LEWIS and SENIOR check	can remove 420 candidates
3) isotopic pattern filter at 5% error	can remove 668 candidates
isotopic pattern filter at 10% error	can remove 632 candidates
isotopic pattern filter at 20% error	can remove 462 candidates
4) H/C ratio check (hydrogen/carbon ratio)	can remove 56 candidates
5) NOPS ratio check (N, O, P, S/C ratios)	can remove 51 candidates
6) heuristic HNOPS probability check	can remove 180 candidates
7) TMS check	can remove 432 candidates

	**combined 10 candidates left**

In order to test a high resolution and high accurate mass instrument, a reference compounds was analyzed by FT-ICR mass spectrometry under robotic nanoelectrospray ionization conditions, Digitoxin (C_41_H_64_O_13_; PubChem CID 441207; 764.939 Da). For Digitoxin, at 2 ppm mass accuracy the seven rules would result still in 146 valid elemental formulas, and at 1 ppm accuracy, still 69 compositions would need to be considered. The experimental mass accuracy for Digitoxin was 0.75 ppm. If isotope errors are lower than 5% relative standard deviation, only 21 formulas would remain, with the correct formula ranked at the 7^th ^position. Isotopic ratio accuracies for Digitoxin were 0.4% (M+1) and 2.4% (M+2). Further reduction of likely compounds can be achieved by screening small molecule databases. Only two formulas were found when screening the 21 possible compositions by the DNP and PubChem databases, with the correct formula C_41_H_64_O_13 _having the higher matching score comparing theoretical and measured accurate mass and isotope data. For this formula, 34 isomeric compounds would be found in the screened databases.

The anticancer agent Paclitaxel (Taxol), (C_47_H_51_N_1_O_14_; PubChem CID 441276; 853.906 Da) was measured on a time-of-flight instrument. Assuming a mass accuracy of 2 ppm (with elements C, H, N, O, P, S, F, Cl and Br) 1418 possible elemental combinations were obtained. Assuming an isotopic pattern error of 3% still 29 formulas were retained, ranking the correct one on the 25^th ^score position. The low rank was mainly due to the influence of fluorine, which is a monoisotopic element and has no impact on the orthogonal isotopic pattern filter. When querying PubChem and DNP databases only one out of these 1418 formulas was found to be known as physically existing compounds, and correctly annotated as C_47_H_51_N_1_O_14 _(Paclitaxel).

The last experiment to test the applicability of the seven rules aimed at low resolution mass spectra. With only 5% isotope ratio accuracy but even worse mass accuracy (> 100 ppm) we determined mass spectra for the natural product solanine (PubChem CID 6326056, C_45_H_73_NO_15_,868.059 Da) using a regular linear ion trap mass spectrometer, which resulted in a measured mass accuracy of 46 ppm; however, calculations were performed at the 100 ppm mass accuracy level. It is notable that the mass accuracy is still better than unit-mass resolution one would expect from an ion trap instrument. Solanine isotope experimental errors were 0.31% (M+1) and 0.08% % (M+1). The program HR2 calculated 7692 possible formulas in three seconds from the neutralized mass spectral data. The seven rules implemented in our Excel script reduced these formulas to abundant 1396 possible candidate compositions assuming a 5% error for isotopic abundances, but after querying PubChem, only twelve hits remained, with the correct formula being ranked top. On a Dual-Opteron PC (2.8 GHz) all steps together were performed in 50 seconds which is an acceptable time for such computations in practice. When testing the DNP library, only two hits were retained, again ranking solanine first. For this example, the set of seven rules removed more than 99.99% of the false formula candidates when combined with PubChem and DNP checks and resulted in the correct compound, despite using low accurate mass data.

## Discussion

The seven rules have generally been developed based on a high cumulative percentage range and this range has ensured that very few existing formulas are rejected as demonstrated by the PubChem validation example. However, our element probability rules have some bias against low mass formulas at the common range (such as methane, CH_4_) for which the rules are too strict, and on the other hand, certain unreasonable formulas like C_23_H_6_O_3 _(which would consist of a very high number of cumulated carbon-carbon double bonds) are allowed by the script. Such senseless formulas need to be sorted out in subsequent steps by scoring the measured isotope ratios or by querying small molecule databases. Developing rules for element ratio checks (rule#4–6) were biased by the development database itself: the Wiley mass spectral database itself was to be unequally distributed. It comprised compounds in the range of 100 Da – 700 Da with a maximum at around 400Da. Hence, more specialized databases might be useful to improve element ratio limits if needed for special applications. For example, the MDL Drug Data Report (DDR) database might be better suited for generating rules for studies involving pharmaceutical drugs. Nevertheless, even the rules developed on the basis of the Wiley and DNP databases turned out to be instrumental as demonstrated by the examination of completely independent PubChem database and specialized examples.

Any positive annotation through database queries must be regarded as preliminary hypothesis and not as ultimate identification. Even if only one substance is retrieved, data might refer to a potential novel compound, since accurate mass and isotope data alone are too weak to positively confirm an individual compound. Instead, further constraints can be added in order to rank formulas according to probability of correct annotations. For example, information on the taxonomy of the species under study may be used. Solanine is a defence compound in potato tuber peels (*Solanum tuberosum*), which would specify the annotation not only to a formula but even to a single chemical compound. The same mass spectral data would lead to an antibiotic tylosin derivate if the sample was derived from *Streptomyces thermotolerans*. Hence, it is useful to utilize background meta-data and additional unrelated (orthogonal) information such as mass spectral fragmentations, volatility or lipophilicity to constrain formulas and rank probabilities for individual compounds. It will further be important to acquire data on a high numbers of pure compounds in order to assess confidence intervals for mass accuracies and isotope errors for specific mass spectrometers, which may then serve as input for lowering the 3 ppm error of mass accuracy and 5% isotope errors that we have taken for calculations in this study. Any such limits will also be dependent on ion statistics, hence the abundance of signals.

The seven rules are currently implemented as an automated script within the EXCEL program, which was particularly useful during development. However, several external programs (MWTWIN, HR2, batch files) had to be embedded causing partial redundancies which could have been avoided if programmed in a single JAVA or C++ application. In addition, EXCEL 2003 or EXCEL XP are incapable of handling more than 65,000 values in one column which is insufficient for mass ranges larger than 2,000 Da when up to several hundred thousand formulas need to be evaluated. The implemented batch function allows an easy check for several thousand single accurate masses from single chromatograms or mass spectral infusion data, if their relative isotopic abundances are included. The bottleneck for more accelerated computations is checking formulas via comparisons of strings. Single compiled files (in C++ or JAVA) could speed up calculations using a binary representation of the molecular formula [50] or other faster database search techniques. Nevertheless, our current formula search implementation is already fast enough for checking 100,000 formulas per second using a binary tree search. The internal molecular formula database is needed, because a direct online check of thousands of formulas would require a substantial amount of time if the service is not optimized for such requests. The directly linked Chemical Structure Lookup Service (CSLS) [51] has the advantage of covering a large space of constitutional isomers (27 millions) and links also to all free and most commercial structure databases which are currently not covered in PubChem. For comparison, the proprietary Chemical Abstracts Service (CAS) currently has 30 million organic and inorganic substances.

We have largely improved the open-source brute-force formula generator HR2 to empower an evaluation speed of 70 million formulas per second. Furthermore, HR2 could easily be linked to open source high speed algorithms for the calculation of isotopic mass and abundance patterns, such as those found in reference [20].

There is a tremendous amount of information on small molecules. However, despite the rapid growth of PubChem, most data were inaccessible for the research presented here because such information is traditionally published in copyrighted (print) journals. So far, this wealth of data is only accumulated in databases by commercial providers such as the Chemical Abstract Service or MDL (Reed-Elsevier, Beilstein) due to the associated high costs of database maintenance and curation. Our approach might have been even more fruitful if molecules, their molecular formulas, molecular properties, spectral data [52], toxicity data [53], taxonomy of investigated species were freely accessible as meta-information which cold be harvested by software robots [54,55] without infringing journal copyrights. Techniques for storing and handling such data are well known since several years and used in the Enhanced open NCI Database Browser [56] and other open-access services. The development of rules for generation of formulas as well as validation efforts would have been even more successful if there were open-access databases of molecular information [57] using the InChI [58] code.

## Conclusion

Development and application of the seven rules has demonstrated that mass spectra are most suitable with a low error for isotopic ratios (1–5%), sufficient resolution (R = 5,000 at m/z 400) and mass accuracy between 1–5 ppm. In fact, mass accuracy was found less important than correct isotope ratio measurements. The most severe remaining bottleneck is validating the initial raw data processing (chromatography peak picking and mass spectral deconvolution) and subsequent determination of adducts to determine neutralized molecular masses. Several algorithms have already been proposed for peak finding, however, further advances on automatic adduct detection must be accomplished [27] in order to cope with the high number of components in complex chromatograms.

The set of seven rules have been shown to correctly annotate accurate mass spectra to elemental compositions for compounds consisting of the elements C, H, N, S, O, P, F, Cl and Br up to 2000 Da, if results are ranked by queries against databases of known molecular formulas. When specialized target libraries are used (e.g. for drugs, metabolites or toxicants), the correct identification rate can be as high as 98%. For novel formulas that are not included in these libraries, the correct elemental composition will be among the top three matches at a probability of 65%. As a rule of thumb, the ranking function alone works well up to 500 Dalton, but at higher masses a small molecule library is needed for correct annotation. Additionally, the seven rules successfully restricted the molecular formula space (less than 2000 Da consisting of the elements C, H, N, S, O and P) from 8 billions down to 623 million formulas. Such a restriction is important for the subsequent database search of corresponding structural isomers [46]. Specifically, this is the first algorithm that calculates formulas with maximal or mixed valence states of elements such as sulphur or phosphorous. The software scripts and programs, source code and all supplement development data are freely available from the Fiehnlab projects site [59].

## Methods

Molecular formulas for the development of the seven rules were taken from the Wiley and NIST02 mass spectral database and the Dictionary of Natural Products. Roughly 47,000 formulas were extracted using the NIST-MS-Search program [25] with the sequential constraints search. Almost 42,000 formulas were retained after excluding compounds that comprised additional elements other than C, H, N, S, O, P, F, Cl, Br or Si. The Chapman & Hall/CRC Dictionary of Natural Products Database (DNP) containing 170,000 single parent entries was accessed via a web interface [60] and purchased as ASCII and SD file version containing all molecular information and meta-data in an Oracle dump file from Informa PLC. The DNP contains data over 200,000 small molecules which can be divided into 80,212 natural compounds, 20,079 drug compounds, 30,470 carboydrates and 33,009 inorganic compounds and other organic compounds (with certain degree of overlap). 31,097 unique elemental compositions were derived from this database. Combining the Wiley and DNP molecule data resulted in 68,237 unique formulas.

The largest publicly available repository of molecular formulas is the NIH PubChem Database [61]. The PubChem database containing 5.3 million compounds was downloaded (search date February 2006) and converted with ChemAxon's free MolConvert tool from SD format to the SMILES structure format [62]. Many other specialized databases like ChemDB [63] and ZINC [64] are now incorporated in PubChem and can be used if chemical property data or information about the commercial availability is needed. The data file was then filtered with regular expressions to remove charged species, salts or isotope-labelled compounds and only allow the elements C, H, N, S, O, P, F, Cl, Br and Si with the free qgrep tool from the Windows Server 2003 Resource Kit Tools [65]. This filter was applied with the constraint that C and H must be present in the formula. Subsequently, the regular expression search step was applied that resulted in a file with 4 million single structures. Exact masses and formulas of these compounds were calculated from the SMILES string using the cxcalc tool from the ChemAxon JChem package v3.1.4 academic version [62]. The result file was sorted according to molecular mass and duplicate formulas were automatically removed by TextPad queries [66]. SDF fields were extracted from the PubChem database with the freely available SDF toolkit [67]. Additional formula searches were performed using the MDL Crossfire Commander and the Beilstein Database and the Chemical Abstracts Database (CAS) and SciFinder Scholar (allowing only one single formula search at a time).

All statistical analyses were performed with Statistica Dataminer v7 [68]. The script comprising the seven rules was developed in Microsoft EXCEL 2003 and most functions were implemented in Excel's Visual Basic macro language. MWTWIN v6.39 was used for the calculation of isotopic abundances and accurate masses within the EXCEL script. The functions were accessed via DLL references from the MWTWIN program which is needed for extended functionality. The EXCEL workbook provides a sheet for manual adduct removal containing 47 adducts for positive and negative ion mode. For high mass accuracy calculations also the mass of the electron has to be taken into account. All calculations require the accurate mass of the neutral form of molecule and the relative isotopic abundances which are normalized to 100%. The core EXCEL script may be used in two ways: (1) for testing the validity of input formulas in the 'controller' sheet by calculating RDBEs, accurate masses, isotopic distributions, element ratios and element probability ratios, Senior and Lewis rules and reporting the result of these checks for each of the formulas by YES/NO outputs or (2) in automated batch mode by entering measured accurate masses and isotopic abundance errors which are then checked and reported by the controller tool. Isotopic abundances must be always entered as relative abundances, normalized to 100% for the highest M+n. Using the experimental isotope ratio data the score-function ranks the results between 0 (no match) to 100 (complete match). This score function adds the differences between the computed and experimental target intensities for each of the M+1, M+2 and M+3 peaks and matches the sum of these differences against the target intensities. The formula generation is done by calling HR2 in an external process. Subsequently, all molecular formulas that are valid within a given isotopic abundance error are checked against an internal database. This internal table contains 432,968 molecular formulas from the PubChem database, including most of the commercially available chemicals and many natural products and covered more than 5 million unique compounds at the time of download. Additionally 120,000 molecular formulas comprising all combinations of the 20 proteinogenic amino acids for masses below 1000 Da and a database of small molecules were downloaded from source [44] and included into the program. User databases or extended newer databases can be very easily updated on demand.

For linking the ranked molecular formulas to structure databases we currently implemented a web reference to the Chemical Structure Lookup Service (CSLS) [42] developed by the Computer Aided Molecular Design (CADD) Group at the National Cancer Institute (NCI) and ChemNavigator.com, Inc, which covers more than 27 million unique structures from 80 databases. Such an approach is possible using SOAP XML [69] or ENTREZ [70] from Pubchem. Other special databases like the Dictionary of Natural Products can be accessed in-house via web services using ChemAxon's JChem or Instant-JChem [71].

For the brute-force calculation of all molecular formulas in the range up to 2000 Da, the program HiRes MS version "20050617" [34] was downloaded and enhanced to HR2. The freely available Microsoft Visual C++ 2005 Express compiler [72] was used for program development. The modified HR2 version can be used to either calculate molecular formulas of an exact mass at a certain mass accuracy (ppm) or it can be used to calculate all formulas in a given mass range. Additionally a faster counting only version can be used to calculate the possible numbers of formula candidates. This is helpful because the output of large formula ranges can result in file sizes of several gigabytes. For calculating elemental compositions the maximum valence values for all elements were used. Element ratio check and element probability check were implemented in HR2. Our current improved version of the brute force formula generator HR2 has a performance of 50–70 million formula evaluations per seconds, depending on the dataset.

Accurate GC-MS mass measurements were performed by time-of-flight mass spectrometry under chemical ionization as published previously [49]. Accurate mass data and accurate isotopic pattern data for Paclitaxel (Taxol) (CAS: 33069-62-4; C_47_H_51_NO_14_; MW = 853.33094) was obtained by using infusion into a time-of-flight mass spectrometer (Bruker Daltonics MicroTOF) with electrospray ionization. The accurate measured mass [M+H]^+ ^was 854.3376 Da (-0.7 ppm error), the measured isotopic pattern were [M+1] = 56.4%, [M+2] = 16.5%, [M+3] = 2.9% with a maximum absolute error of 3.9%. Additional accurate electrospray mass measurements were acquired on a hybrid linear ion trap/Fourier transform ion cyclotron resonance mass spectrometer (ThermoElectron LTQ-FT, Waltham, MA). Pure standards were infused with an automated chip-based nanoelectrospray source (Advion Biosciences NanoMate, Ithaca, NY). At low mass accuracy and low mass resolution, the linear ion trap was operated without using the FT-MS option. The LTQ-FT was calibrated for accurate masses in positive mode using the vendor's calibration mixture. Signal intensities were optimized on each of the substances in autotune mode. The mass range was set from 500–900 Da (widescan mode) and a mass resolution of 50,000 at m/z 400 was specified. A stock solution of 50 μg/ml of Digitoxin (CAS: 71-63-6; C_41_H_64_O_13_; MW = 764.43467) and Solanine (CAS: 51938-42-2; C_45_H_73_NO_15_; MW = 867.49799) was prepared and injected in positive mode with a gas pressure of 0.3 psi and a voltage of 1.6 V by the NanoMate injection system. For each infusion nanoelectrospray mass spectrum, ten mass spectra were averaged by the XCalibur software and transferred to an EXCEL sheet. For calculating adduct ion masses [26] the ESI-MS adduct calculator was downloaded from [73]. In all cases an abundant [M+H]^+ ^adduct ion was detected and the neutralized molecule was used for calculations at a 2 ppm mass accuracy level and for solanine at the 100 ppm level (running the LTQ without FT option) using the program HR2 after conversion from ppm to mmu mass tolerances. For the measured mass of 867.538204 Da for solanine, 100 ppm tolerance refers to 86.75 millimass units. Match tables using the seven rules were prepared assuming a 5% error for isotopic ratio measurements.

All transformations and calculations were performed under Windows XP on a MonarchComputer Dual-Opteron 254 (2.8 GHz, 2.8 GByte RAM), equipped with an Areca ARC-1120 Raid-5 array. This equipment enabled hard disk burst read-write transfer rates of more than 500 MByte/s. An additional RamDisk (QSoft Ramdisk Enterprise) was used for file based operations allowing burst read-write rates of 1000 MByte/s.

## Authors' contributions

Both authors contributed equally to this work and read and approved the final manuscript.
